# Furuncular Myiasis Secondary to Dermatobia hominis Diagnosed With Point-of-Care Ultrasound in the Emergency Department

**DOI:** 10.7759/cureus.74323

**Published:** 2024-11-23

**Authors:** Eric Boccio, Jheanelle McKay, David Hooke, Yehuda Wenger, Brian Kohen

**Affiliations:** 1 Emergency Medicine, Memorial Healthcare System, Hollywood, USA; 2 Pediatric Emergency Medicine, Joe DiMaggio Children's Hospital, Hollywood, USA

**Keywords:** dermatobia hominis, furuncular myiasis, human botfly, pocus, point-of-care ultrasound

## Abstract

Furuncular myiasis is a parasitic disease caused by the larvae of *Dermatobia hominis*, or the human botfly, which burrow under the skin causing cystic lesions to develop.

A six-year-old boy presented with multiple scalp lesions. The mother reported travel to Ecuador one month prior. Bedside point-of-care ultrasound (POCUS) demonstrated hyperechoic foreign bodies superficial to the skull with surrounding anechoic rings, edema, and posterior acoustic shadowing. The diagnosis of furuncular myasis was made, and foreign body removal yielded multiple larvae.

In addition to a thorough history and physical examination, POCUS is a useful modality to visualize encysted larvae and facilitate timely diagnosis of furuncular myiasis when evaluating head, neck, and extremity lesions.

## Introduction

*Dermatobia hominis*, or the human botfly, is native to Central and South America [1]. Furuncular myiasis is the cutaneous manifestation of human botfly larvae infestation which occurs during the parasite’s molting phase [2]. Furuncular myiasis affects both local natives as well as tourists traveling to endemic areas. The condition has characteristic dermatologic findings consisting of a firm, furuncular mass that exhibits a central pore. The lesions are typically pruritic and painful, and patients may describe an intermittent sensation of movement under the skin at the lesion site [3]. Ultrasound is useful in the initial diagnostic workup as the encysted structures that develop are easily visualized and characterized. We describe an unusual cause of scalp lesions in a 6-year-old male with recent travel to Ecuador who presented to an urban pediatric emergency department (ED) in the northeast United States. Consent to present and publish relevant findings was obtained from the patient’s mother. This case was previously presented as a clinical image at the 2023 Society for Academic Emergency Medicine Annual Meeting.

## Case presentation

A six-year-old male was seen by his pediatrician two weeks prior to his ED presentation for scalp lesions. He was prescribed amoxicillin and a delousing shampoo for suspected cellulitis versus lice infestation. Symptoms did not improve despite the completion of treatment. An outpatient ultrasound was arranged and showed multiple echogenic nodular lesions measuring from 0.5 centimeters (cm) to 1.2 cm in the long axis diameter across the scalp. The differential diagnosis entertained at the time included lymphadenitis, benign avascular mass, epidermal inclusion cyst, and pilomatricoma, and the patient was started on clindamycin. Due to concerns about an oncologic process, a surgery consultation was placed, and an outpatient biopsy was scheduled.

Four days after the ultrasound and before the biopsy was performed, the patient and his mother presented to the pediatric ED due to worsening symptoms. Multiple new lesions developed across the patient’s scalp which the mother stated expressed blood when compressed. The patient denied fever and reported intermittent pruritus and pain over the lesion sites. The mother reported a history of travel to Ecuador one month prior to symptom onset. The patient had positive contact with mosquitoes on the face and scalp, and mosquito repellant was infrequently used.

**Figure 1:**
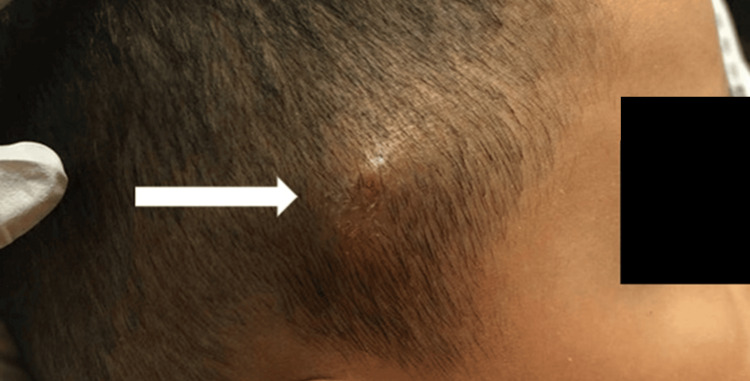
Right temporal scalp lesion. Right temporal scalp lesion measuring 3 by 3 cm in size characterized by induration, erythema, and tenderness to palpation. A small pore was visualized at the center of the lesion with an underlying pulsating fluid level (arrow).

The patient’s initial vital signs were within normal limits: blood pressure at 98/61 millimeters of mercury), heart rate at 73 beats per minute, respiratory rate at 18 breaths per minute, peripheral capillary oxygen saturation at 99% (room air), and temperature at 36.3 degrees Celsius (temporal). A total of six lesions were noted across the entirety of the patient’s scalp, the largest measuring 3 by 3 cm (Figure 1).

**Figure 2:**
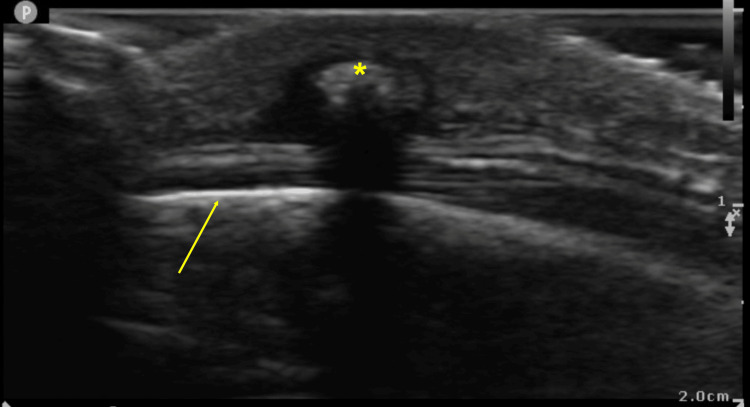
Point-of-care ultrasound findings of right temporal scalp lesion Hyperechoic foreign body (*) superficial to skull (arrow) with surrounding anechoic ring, edema, and posterior acoustic shadowing.

The lesions were raised, indurated, and tender with surrounding erythema. No lesions were identified below the neck or on the extremities. A small, centrally located pore was noted on each lesion, and a pulsating fluid level could be visualized within. No fluid was able to be expressed from the lesions, and there was no associated lymphadenopathy. Point-of-care ultrasound (POCUS) at the bedside using a high-frequency linear transducer was performed (Figure 2).

Given the recent travel history, physical examination, and POCUS findings, the diagnosis of furuncular myiasis secondary to human botfly larvae was made. The outpatient biopsy with general surgery was canceled, and an outpatient referral to dermatology was provided. A review of the patient’s chart revealed a dermatology procedure note describing foreign body removal of a single intact human botfly larva from each of the scalp lesions. The larvae were of various lengths, with the largest measuring 1.2 cm. The patient tolerated the procedure well and was discharged with no sequelae nor return visits to the pediatric ED, per chart review.

## Discussion

*Dermatobia hominis*, known commonly as the human botfly, is native to Central and South America [1]. Human botfly larvae are parasitic and typically grow in the flesh of mammalian hosts. Infestation is facilitated through an intermediate vector, commonly the mosquito, on which a pregnant female botfly will deposit a collection of individual eggs. After a short incubation period, the eggs hatch, and the larvae transfer to the skin of the mammal host on which the vector has landed, usually to feed [2]. The larvae enter the subcutaneous tissue through either the defects caused by the mosquito bite or by burrowing into hair follicles. The larvae feed on host tissue for 27-128 days, dig deeper, and become encysted in the soft tissue. After growing to approximately 5 cm in size during the larval stage, it exits the skin and drops to the ground where it burrows underneath soil or debris to initiate the pupal stage [3].

Human botfly larvae infestation causes furuncular myiasis. Patients typically describe a slow-growing and raised cystic lesion [4]. The lesion is oftentimes pruritic and mildly painful [5]. Patients may state that they detect movement at the site of the lesion under the skin or in their skulls. Fever and lymphadenopathy are rare. The physical exam will reveal a cystic lesion with a central opening containing a pulsating fluid level. If compressed, serosanguinous fluid may be expressed [6-7].

Furuncular myiasis is underreported and often misdiagnosed due to low prevalence in the continental United States [8-9]. POCUS may be useful in the differentiation of skin lesions, and furuncular myiasis has characteristic findings that include a hyperechoic foreign body in superficial and subcutaneous tissues that exhibit shadowing of deeper structures [10-11]. Live larvae will demonstrate movement independent of the host’s positioning which may correlate with patient reports of pruritis, pain, or the sensation of movement under the skin or within the skull.

## Conclusions

Furuncular myiasis is an important diagnosis to consider when evaluating unexplained head, neck, and extremity lesions. In addition to comprehensive history-taking which confirms recent travel to endemic areas and a thorough physical examination, POCUS is a useful modality which can be used to visualize the encysted larvae and facilitate timely diagnosis. POCUS of furuncular myiasis secondary to human botfly larvae infestation will demonstrate a hyperechoic foreign body in the superficial and subcutaneous tissues with posterior acoustic shadowing of deeper structures.
